# Progressive Host-Directed Strategies to Potentiate BCG Vaccination Against Tuberculosis

**DOI:** 10.3389/fimmu.2022.944183

**Published:** 2022-07-28

**Authors:** Kriti Negi, Ashima Bhaskar, Ved Prakash Dwivedi

**Affiliations:** Immunobiology Group, International Centre for Genetic Engineering and Biotechnology, New Delhi, India

**Keywords:** adjunct vaccination strategies against tuberculosis, vaccine, BCG, host directed therapy, immunotherapy, memory T cells

## Abstract

The pursuit to improve the TB control program comprising one approved vaccine, *M. bovis* Bacille Calmette-Guerin (BCG) has directed researchers to explore progressive approaches to halt the eternal TB pandemic. *Mycobacterium tuberculosis* (*M.tb*) was first identified as the causative agent of TB in 1882 by Dr. Robert Koch. However, TB has plagued living beings since ancient times and continues to endure as an eternal scourge ravaging even with existing chemoprophylaxis and preventive therapy. We have scientifically come a long way since then, but despite accessibility to the standard antimycobacterial antibiotics and prophylactic vaccine, almost one-fourth of humankind is infected latently with *M.tb*. Existing therapeutics fail to control TB, due to the upsurge of drug-resistant strains and increasing incidents of co-infections in immune-compromised individuals. Unresponsiveness to established antibiotics leaves patients with no therapeutic possibilities. Hence the search for an efficacious TB immunization strategy is a global health priority. Researchers are paving the course for efficient vaccination strategies with the radically advanced operation of core principles of protective immune responses against *M.tb*. In this review; we have reassessed the progression of the TB vaccination program comprising BCG immunization in children and potential stratagems to reinforce BCG-induced protection in adults.

## Introduction

Tuberculosis (TB) is caused by the facultative intracellular pathogen *Mycobacterium tuberculosis* (*M.tb*). Since the 1800s, TB was the leading cause of health menace worldwide. Despite being declared a global health emergency in 1993 by World Health Organization (WHO), TB continues to be the leading cause of morbidity and mortality amongst bacterial infections (1). The majority of individuals remain asymptomatically and latently infected with *M.tb* owing to confiscation of the pathogen by immune cell populations and this does not lead to disease. Upon serious immunosuppression, around 10% of latently infected individuals develop active TB. Owing to the indefinability of the disease, explosive TB epidemics are hardly encountered which results in underestimated harm caused by *M.tb* worldwide (2). The host immune responses can restrict the pathogen but fail to accomplish complete bacterial sterility. The overwhelming progression of the development of new therapeutics and the emergence of resistant pathogenic strains can be prevented by the enhancement of population-wide immunity against *M.tb* (3).

*M.tb* was originally identified in 1882 by Dr. Robert Koch as the causative agent of TB however, it has lurked among living beings since ancient times. 139 years post-discovery of this pathogen, it still endures as an eternal scourge ravaging globally. Current strategies fail to control TB, due to the upsurge of multidrug-resistant strains, increasing incidents of co-infections in immune-compromised individuals, and the emergence of TB-IRIS (Immune Responsive Inflammatory Syndrome). Globally in 2020, approximately 10 million people were disease-ridden with TB and an aggregate of 1.3 million lives were lost (together with 208000 people with HIV). Furthermore, almost one-fourth of humankind is infected asymptomatically (latently) with *M.tb*, with a 5-15% risk of progressing into clinical manifestations (1). An effective vaccine is indispensable to enhance population-wide immune protection and reduce disease burden. Vaccines operate by stimulating a cascade of immunological responses and ensuing the institution of immune memory against subsequent infections (4). Immune memory was originally described by the Greek historian Thucydides while observing survivors of the plague of Athens and comprehended that survivors have conferred life-long resistance to disease. Hence, he stated that “this disease never took any man the second time” (5). This feature of the immune system is a requisite evolutionary trait. Since historic eras for infectious diseases like smallpox, it is distinctly demonstrated that while initial infections had a fatality rate of 20% to 60% subsequently affected individuals were eternally immune to infection. Edward Jenner was the first to employ this hallmark feature of immunity to treat smallpox and provided a foundation for the development of vaccines (6). Secondary immune responses are refined protective responses mounted by sub-populations of memory cells on subsequent encounters which impart endurance to combat recurrent infections caused by pathogens in the environment. A series of events following primary exposure establishes a pool of long-lasting antigen-specific immune cells that mount quantitatively and/or qualitatively improved immune response upon reinfection. Documented from the times of ancient Greeks but still, many components of immune memory are still debatable (5). Immune memory is the cardinal property of the adaptive immune system and exclusively lymphocytes were known to mediate these responses. However, organisms that lack T and B lymphocytes similarly possess heightened proficiency to combat recurring infections caused by the same pathogen, demonstrating the existence of innate immune memory (7). Numerous studies in simpler living beings have reported that cells of the innate immune system can mount heightened secondary responses upon reinfection. Thus, the conventional characterization of immunological memory is continuously advancing (8). Better insights into the generation of immunological memory are fundamental to foster progressive vaccination strategies.

Despite pre-exposure vaccination with BCG, a large extent of latent TB infections urges the need for an efficacious complementary TB control strategy. It is evident that the most extensively used vaccine, Bacille Calmette-Guerin (BCG) which has existed for 100 years fails to impart long-term immunity and has limited efficacy against adult pulmonary TB (9). BCG immunization has a limited impact on *M.tb* transmission since it cannot inhibit primary infection or recrudescence of latent TB (10). Failure to develop a significantly effective vaccine has constrained the phasedown of the global TB burden (9). Furthermore, safety concerns regarding BCG immunization in immune-compromised individuals including HIV-TB coinfected individuals necessitate a vaccine that is safer and more efficient than BCG and can ameliorate infections (11). The efficacy of BCG against pulmonary TB is even more disappointing in tropical regions with a high TB burden (12). Escalating TB cases worldwide despite BCG administration further demands the advancement of the existing vaccination strategy. In the past decade, various research groups have utilized pioneering technologies to improve the current scenario. Progressive vaccine design strategies have been implemented and vaccine candidates have been evaluated in different clinical trials (13). It is challenging to discover more efficacious vaccine candidates for TB that can substitute BCG. It is highly critical to comprehend the shortcomings and prospects of novel vaccination strategies for better implementation and amendment of TB control measures. **Table 1** summarizes key desirable characteristics of improved vaccination strategy. In the current scenario, COVID-19 and TB are the top two causes of death from contagious diseases (1). The similarity in symptoms, risk factors, and primary organ affected further exacerbates the circumstances. TB-COVID-19 co-infection certainly intensifies the severity and threat of death. Even though both diseases primarily affect the lungs, due to disparities in modes of transmission and pathogenesis, distinct therapeutic measures are required (14). Nevertheless, with extraordinary scientific endeavour, information regarding the pathogenesis of SARS-CoV-2 was channelled to develop diagnostics, therapeutics, and vaccines for COVID-19. Further, years of implementation of TB control programs were utilized to implement control strategies to constrain the pandemic (15). This prompts the necessity to retrospect and harnesses the paramount knowledge for progressive solutions to counteract the syndemic of COVID-19 and TB. Lessons learned from BCG vaccination for TB have been operative to control the COVID-19 pandemic owing to the broad-spectrum immunomodulatory potential of BCG (16). The emergence of Severe Acute Respiratory Syndrome Coronavirus 2 (SARS-CoV-2) and the consequent COVID-19 pandemic has negatively influenced the years of progress in tuberculosis (TB) control (17). Advancement in the direction to put an end to TB was hit hard by the ongoing COVID-19 pandemic. Reports of the World Health Organization (WHO) corroborate that advent of COVID-19 has caused a diminution in TB diagnosis and escan alation in mortality. To augment protective immune responses against TB in adults, massive scientific and economic attempts have been made globally. Aspiration to attain complete bacterial sterility to counteract active TB and restrict the transmission with next-generation vaccines has been actively trailed. Even with several vaccine candidates in different phases of clinical trials, we are yet to uncover vaccination strategies that can efficaciously restrict escalating TB burden worldwide (17). Hence, in this review, we have discussed strategies to augment the existing vaccination approach. Immune responses induced by BCG vaccination have been studied comprehensively in past and we are still uncovering new information regarding host responses stimulated by BCG that impede the establishment of effective memory responses (18). To improve the immunotherapeutic efficacy of BCG it is vital to completely understand the mechanism of BCG-induced immune responses. Modulation of host immunity *via* immunomodulators along with vaccination can be employed as a stratagem to incline immune responses to attain ever-lasting immunity against *M.tb* infections. We will reassess the radical approaches utilized by the researchers to limit the prevailing TB cases and how a better understanding of BCG is prime for progress in the TB vaccination program.

**Table 1:** Desired characteristics for novel Tuberculosis immunization approaches.

S. No	Desired characteristics
1.	Safe to be administered in immunocompromised individuals at risk of developing active TB
2.	Expenses associated with regimen and dosage should be reasonable for high burdened developing countries.
3.	Immunization strategy must lower the risk of developing active pulmonary TB in adults previously vaccinated with BCG
4.	Must protect against *M.tb* infections for more than 10 years subsequent to immunization
5.	Minimum administrations requisite to elicit host protective responses
6.	Evaluation of protective immune correlates by employing established assays
7.	Must offer greater than 50% protective efficacy against established pulmonary TB

## Expedition From Virulent *M. bovis* to the BCG Vaccine:

Albert Calmette and Camille Guérin commenced the pursuit to develop a vaccine against TB in 1900 at the Pasteur Institute (19). They began by cultivating a virulent bovine strain of bacillus which was isolated from a tuberculous cow by Nocard. Initially, the bacilli were grown on glycerine, potato medium supplemented with ox bile to limit the clumping and attain homogenous bacterial suspension. This effort to minimize bacterial clumping additionally lowered the virulence of pathogen upon sub-culturing. This scientific observation provoked the scientists further to focus on using the attenuated strain of bacilli for the generation of TB vaccine (20). Till 1919, they successfully sub-cultured the bacilli more than 230 times. This strain failed to infect and cause TB in animals such as guinea pigs, cattle, and rabbits. Firstly named “Bacille Bilie Calmette-Guerin” this is now the most widely administered vaccine worldwide Bacille Calmette-Guerin (BCG) (20). BCG was first utilized in 1921 to immunize a new-born *via* oral route after which it was mass vaccinated to protect infants from disseminated forms of TB. To justify the escalating demand for vaccine strain worldwide, several laboratories around the world began sub-culturing BCG owing to which individuals around the world are vaccinated with characteristically distinct BCG strains (21). Based on existing knowledge, it is evident that diverse BCG strains have variable efficaciousness (22) and immunogenicity (23) but the most efficient BCG strain is yet to be established (23). As little as 1% augmented efficaciousness can rescue around 18,000 individuals and limit 83,000 TB cases in a year (24). Hence, to better understand the protective co-relates of BCG vaccination should be the top priority to upgrade the vaccination strategy.

## BCG Induced Protection Against TB

**Figure 1:**
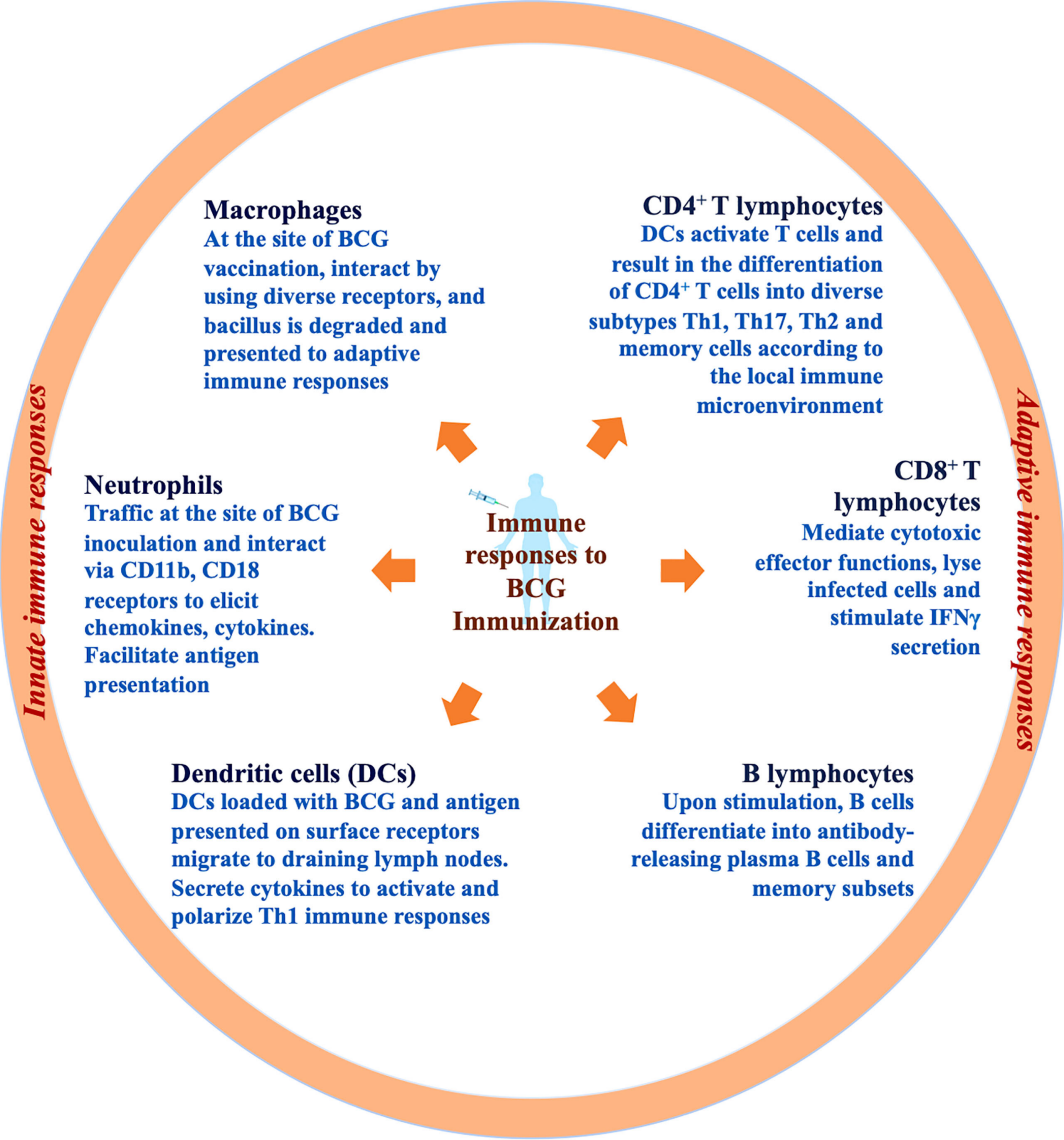
Immune responses to BCG immunization. Immune responses to the BCG vaccine initiate at the site of inoculation by induction of innate immune cells such as resident macrophages, neutrophils, and dendritic cells. Innate immune cells internalize, degrade and present antigen of bacilli *via* surface receptors to further activate adaptive immune cells. Chiefly, DCs loaded with bacilli drain to the lymph nodes and result in lymphocyte stimulation and activation. T and B lymphocytes further differentiate into diverse subtypes including effector and memory cells.

Since the launch of BCG in TB immunization programs, numerous lives have been saved owing to the only existent TB vaccine (19). The percentage decline in disease that can be attributed to vaccination outlines the clinical effectiveness of a vaccine. Mainly, the BCG vaccine is administered to protect against TB. However, the protective efficacy of BCG is assessed distinctively in the case of disseminated TB in children and adult pulmonary TB on the account of colossal discrepancy (25). One of the multifaceted explanations can be the intricate biology of TB establishment and progression in humans (26). In the majority of *M.tb* infections, the immune system can proficiently restrict the progression of the pathogen to cause active TB. However, the endeavour to eliminate the bacilli completely is rarely achieved and can culminate into escalated inflammatory responses that direct distinct phases of disease such as latent TB and associated immunopathogenesis (27). The aim is to strike a balance for the resolution of *M.tb* infection exclusive of detrimental inflammation. For more than a century, researchers have attempted to ascertain correlates of BCG-induced defenses in humans through various animal models (10) and clinical trials (28). Additional to *M.tb* infections, broad-spectrum immunomodulatory characteristics of BCG are utilized to treat bladder cancer (29), asthma (30), leishmaniasis (31) and warts (32). While our understanding of key immunological aspects is continuously expanding (33), we have attained substantial knowledge regarding innate and adaptive immune responses to *M.tb* infection and BCG immunization which is briefly depicted in **Figure 1** and will be reviewed thoroughly in this section.

Innate immune system function as the first line of defense in confronting *M.tb* infections (34). The innate immune responses are the key component of the host immune responses engaged promptly at the site of infection (35). Even in the course of intradermal BCG immunization, early immune responses are elicited by resident epidermal macrophages (36), neutrophils (37), and dendritic cells (DCs) (38). BCG comprises pathogen-associated molecular patterns (PAMPs) such as cellular components (mycolic acids, peptidoglycans, and arabinogalactans) which are recognized by diverse PAMP recognizing receptors (PRRs) present on the surface of innate immune cells. Diverse PRRs abundantly expressed on innate subsets such as complement receptor 3 (CR3) (39), TLR2/4/9 (40), mannose receptor (41), Ca^2+^-dependent lectin (MINCLE) receptors on macrophages (42), nucleotide-binding oligomerization domain (NOD)-like receptors NOD2 on monocytes, CD18, FcγRII, and FcγRIII on neutrophils and DC-SIGN, CD11c, and CD205 on dendritic cells initiate the prompt innate immune responses upon BCG immunization (43). Disparities in the cellular composition of BCG and *M.tb* has been linked with recognition by different PRRs which further influences the uptake, processing and representation of antigens to other immune cells. Since the receptor involved determines the fate of downstream signaling, the variation in receptor utilization can be further associated with relatively inefficient immune responses in the case of BCG (44). Examination of skin biopsies demonstrated that BCG blister point majorly comprises CD15^+^ neutrophils, a small proportion of CD14^+^ monocytes, and an infinitesimal population of CD3^+^ T lymphocytes (45). However, in whole blood culture experiments, CD56^+^ NK cells, γδ T cells, NKT cells, and cells from MAIT were found to be associated with BCG-induced immunity (46). In response to BCG vaccination, innate immune responses such as ROS/RNI generation by neutrophils, the release of monocytic chemokines like IL-6, TNF-α, MIP-1α, MIP-1β, IL-8, and IL-1α within 1-3 hours is initiated to direct systemic immune responses (45). BCG is known to effectively activate monocytic populations (47). In animal models, subsequent to BCG immunization mycobacterial extermination by macrophages was demonstrated independent of adaptive immune responses (48). Deficit immune responses induced by macrophages subsequent to BCG vaccination downgrade bacterial clearance. Furthermore, guinea pigs immunized with BCG upon H37Rv infection demonstrated enhancement of phagosome-lysosomal fusion with a considerable reduction in mycobacterial burden (47).

Dendritic cells (DCs) function as a nexus between innate and adaptive immune responses by presenting processed antigens to T lymphocytes post BCG immunization *via* IL-1R, MyD88 pathway (49). BCG immunization is known to enhance DC maturation and activation by upregulating the expression of markers implicated in antigen presentation such as MHC-II, CD40, CD80, and CD86 (35). Nonetheless, BCG immunization is also linked with the stimulation of IL-10 and IL-4 cytokines by DCs which can bias the differentiation of T lymphocytes toward the T_H_2 subtype and can be the cause of weakened BCG effectiveness (49). Nonetheless, the majority of information concerning the role of DCs in the case of BCG inoculation is from *in vitro* studies. The observation that *in vitro* BCG stimulation, initiates aggregation of DCs, upregulates antigen presentation with reduced endocytosis, and stimulation of TNF-α infers that DCs contribute to the initiation of immune responses. However, it is concerning that compared to *M.tb* infection these responses are inadequate to impart requisite protection (50). Apart from the major innate immune cell populations, innate lymphoid cells (ILCs) and mucosal-associated invariant T cells (MAIT) have been connected with BCG-induced innate protection (36). However, fragmentary information is accessible regarding these subsets and further exploration is necessitated.

It is now well-known that analogous to antigen-specific responses elicited by adaptive immunity, subsequent to pathogenic insults cells of innate immunity elicit heterologous memory responses (7). Distinct reports have demonstrated that natural killer (NK) cells and macrophages which have formerly encountered pathogens through epigenetic remodeling are trained to respond to distinct pathogens (51). It has been observed that epigenetic modifications such as H3K4me1, H3K4me3, and H3K27ac have been associated with the reprogramming of monocytic populations owing to unfastening of chromatin positions at the promoters of pro-inflammatory cytokines (52). BCG immunization leads to the expansion of Hematopoietic stem cells (HSCs), drives myelopoiesis and *via* epigenetic reprograming enhances host protective immune responses (53). Furthermore, it is established that macrophages interact with NK cells and bring about the refinement of innate immune responses against pathogenic insults (54). With a deeper insight into trained immunity, it was observed that BCG vaccination in healthy individuals stimulates NK cells and macrophages to uphold cytokine generation in response to *ex vivo* stimulation (55). The broad-spectrum immunomodulatory potential of BCG has been employed for the treatment of diverse ailments including the COVID-19 disease caused by SARS-CoV-2 (56). It has been experimentally proved that BCG *via* epigenetic reprogramming of immune cells exhibits cross-protection against diverse pathogens (57). The research demonstrates the impact of BCG vaccination on the induction of genome-wide histone modifications in trained monocytes which participate in IL-1β generation and the reduction of the yellow fever virus (YFV) burden (58). BCG-induced protection against YFV infection substantiates the broad-spectrum effectiveness of the vaccine against diverse viral infections such as influenza A (H1N1) virus, herpes simplex virus (HSV), and human papillomavirus (HPV) (59). Based on this information, BCG vaccination was evaluated initially during the COVID-19 pandemic. Preliminary ecological studies demonstrated that COVID-19 cases and deaths per population were fewer in countries with BCG vaccination schedules (60). The notion of inducing anti-viral immunity by employing BCG was based on the generation of heterologous immune responses (61). Since it was found that the envelope protein of the SARS-CoV-2 virus shared certain homology with strains of *Mycobacterium* species (62). It was inferred that the homology was associated with the induction of host-protective T_h_1/T_h_17 responses. The concept of trained immunity and heterologous responses were utilized to exploit BCG vaccination in the era of COVID-19. Since the majority of individuals are already vaccinated with BCG, it is judicious to keep BCG in reflection while developing new vaccination strategies. The fight to halt TB must continue progressively while dealing with the ongoing COVID-19 pandemic (17).

Till now adequate information is established regarding protective correlates against TB. Adaptive immune responses play a vital role in eliciting pathogen-specific immune responses with superior efficacy (26). The protective role of T lymphocytes was primarily demonstrated by the adoptive transfer of CD4^+^ and CD8^+^ T cells from BCG immunized mice to T and B cell-deficient (Rag1-/-) knockout mice (63). T lymphocytes contribute significantly against *M.tb* infections upon activation by components of innate immunity. The induction of T_h_1/T_h_17 immune responses and IFN-γ secretion is positively linked with augmented clinical outcomes in TB patients (26). Several studies have demonstrated mechanistic insights of BCG-induced defenses as a consequence of T_h_1 cells through IFN-γ secretion (64). The paramount contribution of T_h_1 responses was also demonstrated in infants vaccinated with BCG wherein T_h_1 responses prevailed for more than a year contrary to pronounced T_h_2 responses in unvaccinated infants (65). Furthermore, for the next few years, BCG-induced protection was attributable to IFN-γ releasing T lymphocytes (65). However, the outcomes of BCG immunization are still disputable and not strongly concurrent with specific immune responses. In IFN-γ deficient mice, BCG immunization demonstrated considerable protection against *M.tb* infection that vanished after depletion of CD4^+^ T lymphocytes (66). Comparable outcomes were achieved in a study involving humans vaccinated with BCG wherein restricted *M.tb* progression and protection were linked with IFN-γ independent CD8^+^ immunological responses (67). It is established now that polyfunctional CD4^+^ T lymphocytes play a vital role in enhancing defenses against *M.tb* by secreting cytokines in different combinations to amend the microenvironment at the site of infection (68). It was confirmed that BCG immunization in infants does not elicit polyfunctional immune responses linked with effective protection against *M.tb* (12). However, immunization with a booster dose of MVA85A (modified vaccinia virus Ankara expressing antigen 85A) in BCG vaccinated adults confirmed induction of polyfunctional T cell responses (69). This gave rise to the hypothesis of heterologous boosting of BCG immunization for robust protective immunity against *M.tb*. Thereafter, failure of MVA85A heterologous boosting in infants to induce efficacious immune responses even with induction of polyfunctional T responses blurred the resolution of protective efficacy (70). Similarly, CD8^+^ T cells exhibit antimycobacterial activity and are directly involved in *M.tb* killing, cytotoxic extermination of infected cell populations, and IFN-γ secretion (71). In human samples, CD8^+^ T cells have been shown to distinctively identify and kill infected macrophages along with internalized *M.tb* with granular discharge comprising perforin and granulysin (63). Therefore, subsiding the bacterial burden and effectively enhancing defenses against *M.tb*. The vitality of MHC class I-restricted CD8^+^ T cells was demonstrated in β2-microglobulin (β2m) deficient mice incapable of restricting *M.tb* infection (72). It is not feasible to achieve sterilizing immunity against reinfections with mycobacteria subsequent to pathogen clearance with antimycobacterial drugs. Several studies have emphasized the significance of IL-17 generating CD4^+^ T lymphocytes in mediating protection from reinfections and improving clinical outcomes upon vaccination (73). Consistent with these reports, in BCG immunized mice, T_h_1 responses in the lungs were shown to be reliant on IL-17A and IL-23 secreted by antigen-specific T_h_17 cells (73). In non-human primates (NHPs) administering a high dose of intradermal or intravenous BCG was linked with augmented protection from *M.tb* as a consequence of CD4^+^ T lymphocytes with T_h_1/T_h_17 phenotypic characteristics (74). Thus, augmenting T_h_1/T_h_17 responses induced by BCG offer prospective solutions against TB (74). BCG immunization has been linked with the enhancement of regulatory T cells (T_regs_) *via* alteration of immune metabolic pathways which consequently reduces the protective efficacy against TB. Depletion of T_regs_ can result in enrichment of T_h_1, cytotoxic T cell responses with enhanced bacterial extermination upon infection (75). Boosting BCG with a novel vaccine candidate comprising Ag85B-Mpt64 (76–84)-Mtb8.4 (AMM) along with composite adjuvant lowers the T_reg_ population which was associated with enhanced protection in the mice model (85). Nonetheless, few studies have also demonstrated unaltered outcomes in BCG immunization upon prior T_regs_ depletion (86). Hence, further analysis is necessitated to validate the prospects for utilization in clinical operation. Regardless, the potential of the BCG vaccine to enrich T_regs_ and inhibit detrimental inflammation has been employed to treat diverse disorders including SARS-CoV-2 infection-induced cytokine storm in the COVID-19 pandemic (87). Additionally, BCG administration has been linked with increased IL-10 secretion in animal models which consequently restricts anti-mycobacterial pro-inflammatory responses induced by vaccination (88). Furthermore, obstruction of IL-10 signal transduction in the course of BCG immunization improved protective immune responses by mounting T_h_1, and T_h_17 responses (89). Hence, striking the immunological balance to achieve affirmative outcomes is requisite to alleviate BCG-induced protection.

## Approaches to Amend Inadequacies of BCG Vaccine

**Figure 2:**
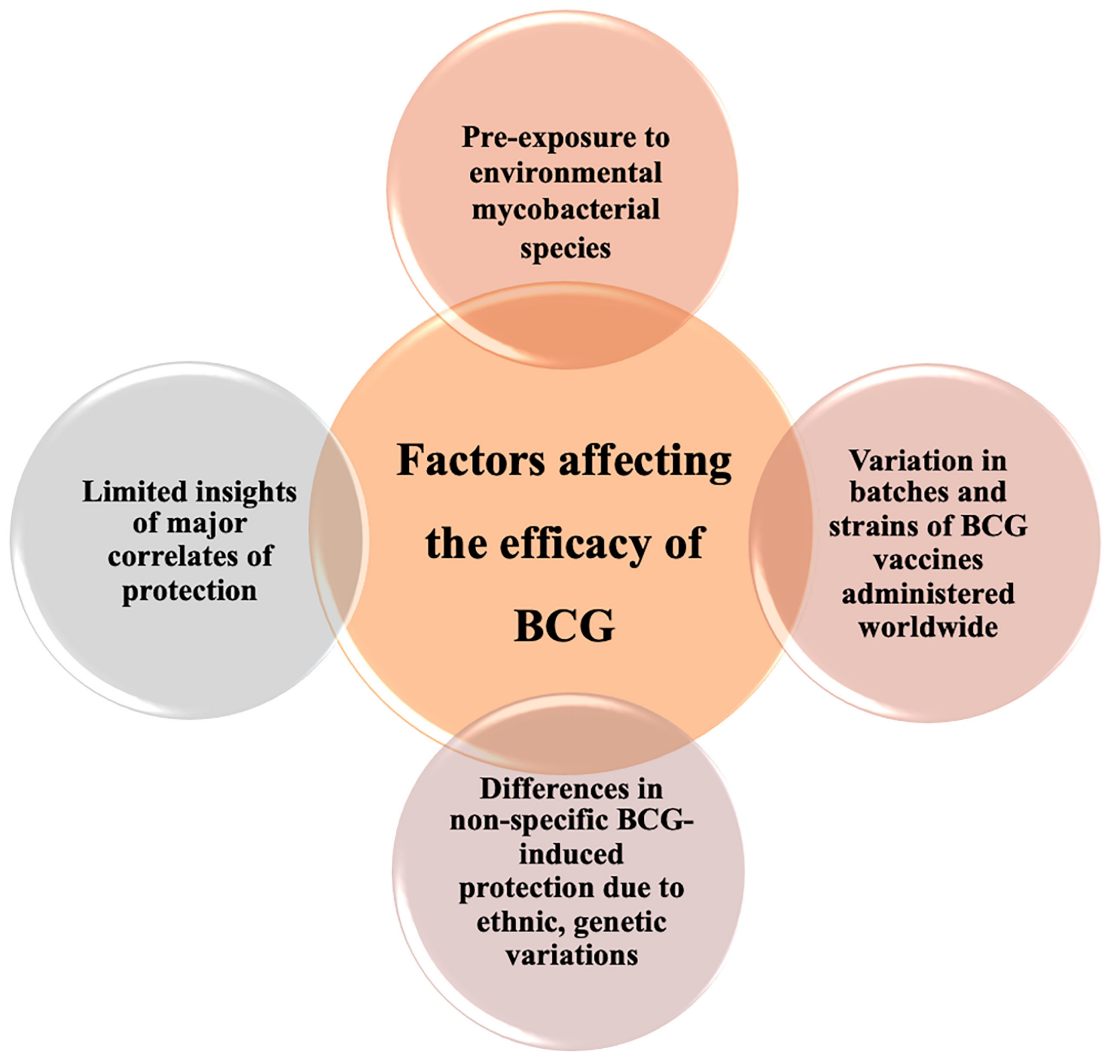
Diverse factors associated with variable efficacy of BCG. Inconsistent efficacy of BCG vaccination can be linked to numerous host factors including genetics, geographical representation, ethnicity, and fragmentary immunological insight addition to variation in BCG vaccine strains with distinct characteristics.

Despite numerous limitations of the BCG vaccine, it is still challenging to stumble upon more effective vaccine candidates for TB (90). BCG is unquestionably the most reliant vaccine for the prevention of disseminated forms of TB in children (91). Hence, it is critical to comprehend the shortcomings of BCG to extemporize protection by progressive approaches. It is widely proclaimed that wearying BCG immunity is a consequence of the non-existent T cell epitopes of *M.tb* in BCG vaccine strains (92). It is also established that expansion and differentiation of effector T cells declines in age-dependent manner upon BCG inoculation inferring toward weakened central memory responses (93). The contracted pool of antigen-specific memory populations is the basis for short-term protection against *M.tb* infections (93). Furthermore, the variable efficaciousness of BCG-induced defenses is associated with multifold factors such as ecological aspects, genetics, and differences in nutritional profiles amongst populations, listed in **Figure 2**. A major rationale for highly variable BCG efficacy in adults is exposure to environmental non-tuberculous mycobacteria (NTM). Prevalence of NTMs in tropical regions has been linked with low efficacy of BCG and consequent high TB burden due to pre-immunization exposure induced variation in protective efficacy (94). In the regions nearby the equator, UV exposure has been connected with a reduction in BCG efficacy which was further demonstrated in animal models with alterations in cytokine profiles when exposed to UV at the time of BCG immunization (60). Furthermore, variations in handling protocols and numerous passages of BCG vaccine strains have given rise to alterability in immunogenicity of BCG vaccine strains worldwide (95). Despite the wavering protective efficacy of BCG vaccine is deemed to be safe and is administered worldwide is numerous vaccination programs. However, with the raising concerns in immune-compromised HIV-TB co-infected individuals, WHO has addressed disputes regarding the utility of live vaccine in diverse risk groups. In immune-deficient children seldomly BCG vaccination can lead to systemic BCGosis. Atypical adverse reactions were detected in children with chronic granulomatous disease, Di George syndrome and severe combined immune deficiency (SCID) which can result in deadly consequences if unmanaged (96). In immunocompromised individuals especially neonates vaccination can also result in BCG lymphadenitis and disseminated BCG infection which is one of the most detrimental consequence of BCG vaccination (97). Hence, due to the overabundance of factors contributing to undermining protection elicited by BCG progressive approaches have been employed to improvise BCG against *M.tb*.

## Replacing or Reclaiming BCG

The major TB burden worldwide accountable for morbidity and mortality is due to adult pulmonary TB cases (1). The interval of weakening of BCG-induced defenses overlaps with an escalated incidence of *M.tb* infections in adults. On the surface foremost justification for the incompetence of the BCG vaccine appears to be immunization in the early years of life which imparts limited protection (90). The prospects of the End TB Strategy hence seem bleak without an improvised vaccination stratagem. Since BCG is the most widely utilized vaccine in the world and imparts protection in infants against disseminated forms of TB, it is judicious to reclaim BCG-induced protection rather than displacing it with a replacement. Fundamental strategy to amend BCG efficacy is by utilizing prime boost vaccination approach (98). Since one of the many desired characteristics for upcoming vaccine candidates is to efficaciously improvise the existing TB control approach i.e. prophylactic BCG immunization (99). Alternatives to strengthen the existing TB control program comprises developing a booster vaccine to augment the protective efficacy of BCG or supplementing immunotherapeutic as an adjunct to strengthen BCG-induced immunity. Our research group has endeavored and designed a novel vaccine comprising TLR2 and TLR9 agonist along with collective of 7 overlapping immunogenic *M.tb* peptides, packed together in a liposome (PTL). We have demonstrated that intranasal immunization with PTL along with BCG drastically condensed the bacterial burden, enhanced host protective T cell responses with expansion of polyfunctional T cells as well as memory T cell subsets. Furthermore, host protective immune responses were *M.tb* specific owing to which spectrum of host protective signaling pathways critical to control TB were activated in response to PTL BCG co-immunization (100). Further assessment in higher animal models like Non-human primates (NHPs) is coveted to further validate our findings and progress the research to higher phases. We and various groups worldwide have actively devised and pursued strategies to develop new vaccine candidates, booster vaccines and immunotherapeutic to augment BCG efficacy against *M.tb* infection. However, in this review we have discussed about new TB vaccines in brief and have focused on host immunotherapeutic approaches comprehensively.

## New Vaccines Against TB

The necessity of an alternate vaccination strategy for TB control has not been overlooked and researchers around the world are actively pursuing diverse vaccine candidates to improve existing circumstances (99). On the account of immense attempts, several groups have developed vaccines by employing diverse approaches to control TB. Some classic examples include the attenuated *M.tb* bacilli strains (101) with high immunogenicity (102), the generation of genetically modified BCG strains for better immune responses (70), sub-unit vaccines incorporating immunogens absent in BCG (103), and adjuvants with enhanced potency. Major TB vaccine candidates in advanced clinial trials are tabulated in **Table 2**. A myriad of challenges is liable for slow progress in vaccine development against TB. One of which is the necessity for a vaccine that protects against adult pulmonary TB, in individuals who are presently vaccinated with BCG. Ideally, the development of vaccine candidates that can efficaciously impart protection to individuals previously exposed to mycobacteria including BCG, *M.tb* and environmental mycobacterial species would be enviable. So as to boost the immune responses thereby limiting adult pulmonary TB infections (19). Diverse studies incorporating booster vaccinations are under evaluation in animal models (104). With the advancement in technology and knowledge regarding host protective defense mechanisms, especially antigen-specific immune responses accountable for effective responses against *M.tb* we are surpassing conventional vaccination approaches. With improved understanding regarding known correlates of protection, research groups are assessing strategies to enhance long-lasting protective immune responses by employing progressive targets. What we have learned in the past decade from BCG trials as well as recent COVID-19 trials can be employed in the future to better interpret the lacunae which can be resolved for an improved TB vaccination program (16).

**Table 2:** Major TB vaccine candidates in clinical trials:.

Vaccine candidate	Composition	Clinical Trial	Clinical trial Identifier	Ref.
**Inactivated whole-cell vaccines**				
DAR-901	Inactivated *Mycobacterium obusense*	Phase 2, randomized, placebo-controlled, double-blind study to evaluate the efficacy of DAR-901 TB booster to prevent TB in adolescents.	NCT02712424	(1)
MIP	Inactivated *Mycobacterium indicus pranii*	Phase 3, randomized, double-blind, interventional study to determine the efficacy and safety of MIP as an adjunct in Category I pulmonary TB patients	NCT00341328	(2)
RUTI^®^	Detoxified, fragmented *M.tb* contained in liposomes	Phase 2, randomized, double-blind, placebo-controlled interventional trial to assess the therapeutic vaccine, RUTI against TB	NCT01136161, NCT04919239	(3, 4)
Vaccae™	Heat-inactivated *Mycobacterium vaccae*	Phase 3, randomized, double-blind, interventional trial to assess the safety and efficacy to prevent TB in high-risk groups of TB infection	NCT01979900	(5)
**Live attenuated vaccines**				
MTBVAC	Live attenuated *M.tb* vaccine with PhoP and FadD26 deletions	Phase 3, randomized, quadruple masking intervention to determine safety, efficacy and immunogenicity in newborns	NCT04975178	(6)
VPM1002	Live recombinant BCG vaccine strain with urease C deletion engineered to express listeriolysin rather than urease C	Phase 3, multicenter, double-blind, randomized, active-controlled trial to examine the safety, efficacy and immunogenicity to prevent *M.tb* infection	NCT04351685	(7)
**Subunit vaccines**				
M72/ASO1E	Fusion protein subunit vaccine based on 32A and 39A prepared in AS01_E_ adjuvant	Phase 2, randomized, interventional clinical trial to determine the efficacy of TB vaccine candidate in Adults	NCT01755598	(8)
H56:IC31	Recombinant vaccine comprising proteins of *M.tb* (85B, ESAT6, Rv2660c) and IC31 adjuvant	Phase 2, randomized (1:1), double-blind, placebo-controlled trial to determine efficacy of H56:IC31 in preventing rate of TB recurrence	NCT03512249	(9)
GamTBvac	Recombinant subunit vaccine formulation comprising modified Ag85a and ESAT6-CFP10 *M.tb* antigens and CpG ODN adjuvant	Phase 3, randomized, multicentered, double-blind, placebo-controlled intervention to determine safety and efficaciousness of GamTBvac against pulmonary TB	NCT04975737	(10)
ID93/GLA-SE	ID93 is a recombinant fusion protein comprising 4 antigens from virulence-associated proteins in GLA-SE i.e., oil-in-water emulsion	Phase 2a, randomized, placebo-controlled, double-blind intervention to evaluate safety and effectiveness of ID93/GLA-SE in TB patients	NCT02465216	(11)

## Host-Directed Strategies to Improve BCG Efficiency

The outcome of *M.tb* infection is determined not just by the action of the pathogen but also by the host response. So as to achieve the goal of the End TB strategy, progressive efforts are being made to establish efficacious therapeutics (17). In an attempt to achieve this goal, host-directed therapeutics with the potential to reprogram host defenses for better clinical outcomes are under consideration. Since it is known that in the majority of individuals, the immune system can self-reliantly eradicate the pathogen. Augmenting this phenomenon so as to achieve complete sterility can offer benefits to the existing global TB burden. TB is a chronic disease with a spectrum of pathologies (26). Hence, we need to move ahead from conventional antibiotics toward host-directed therapies for improved clinical outcomes. HDT has found a niche in the treatment of various diseases (105). However, we need more efforts to establish a standard HDT as an adjunct to conventional ATT for the augmentation of disease burden. So as to achieve this target it is a prerequisite to determine key host immune targets for better outcomes. HDTs aim at diverse pathways critical for determining the fate of the infection. HDTs work by restricting pathways exploited by pathogens or by ameliorating host protective immune responses (105). With the advancement in understanding, diverse factors contributing to the establishment of infection have been identified. The primary goal of TB drug discovery is to exterminate both active and persistent bacteria so as to attain complete sterility. Challenge is to aim at heterogenous *M.tb* populations that respond distinctly to therapeutics. It is essential to be reminiscent of the fact that *M.tb* infection can instigate a continuum of host responses owing to distinctive physiologies of heterogeneous bacterial populations. Furthermore, a profound assessment of stochastically and phenotypically drug-resistant persisting populations of *M.tb* subsequent to drug therapy is requisite to cope with TB relapse and reactivation (106). Better insight into mechanisms targeted to exterminate persistent populations is necessitated since it is not conclusive whether targeting bacterial membrane or prime respiratory components will eradicate latent bacteria. It is the need of the hour to get hold of innovative drugs to establish efficacious therapy to attain the goals of the End TB strategy. To accelerate the search for the right drugs, United States Food and Drug Administration (FDA) approved compounds are also under evaluation and operation (107).

**Figure 3:**
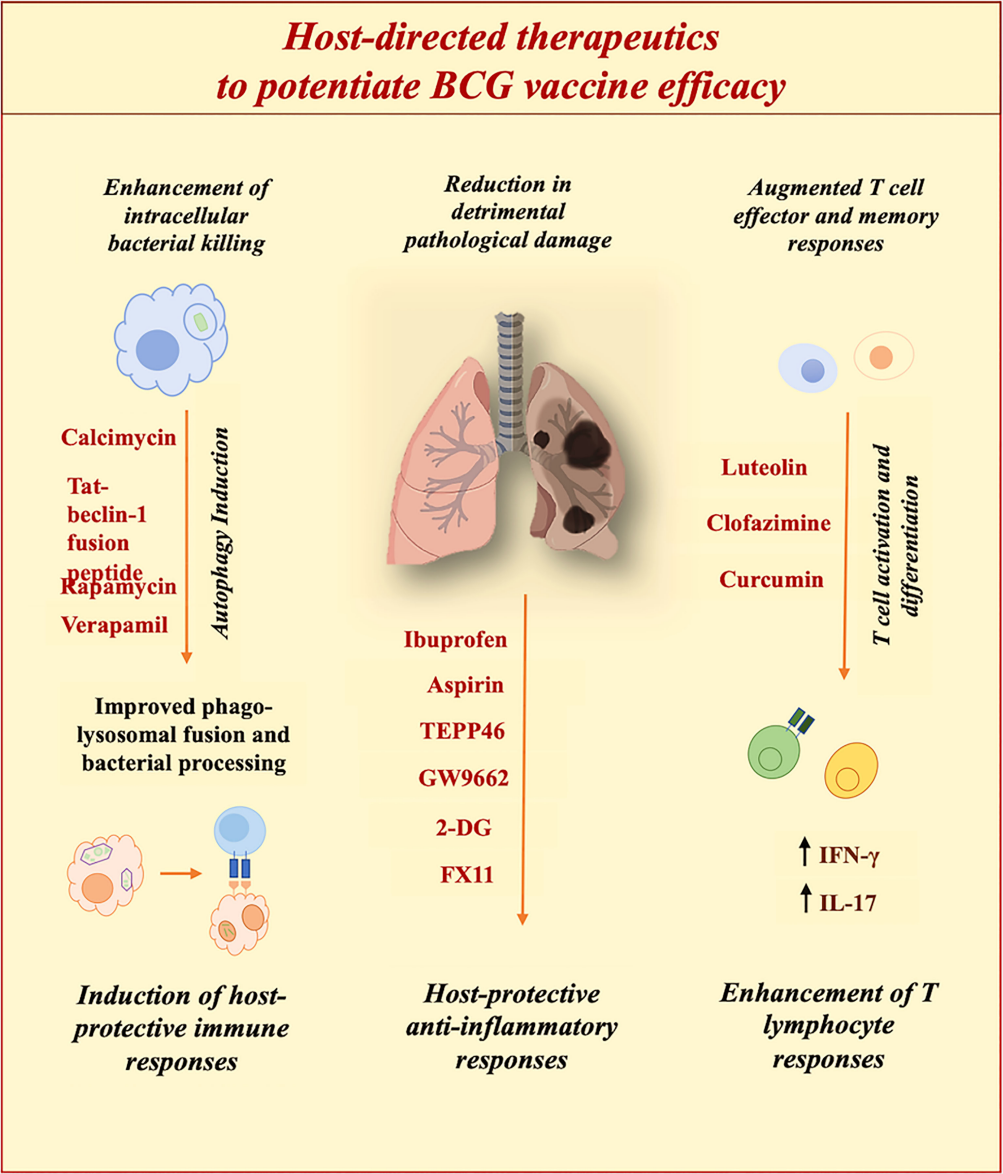
Potential of host-directed therapies (HDTs) to improve BCG efficacy. Diverse HDTs aiming at distinct pathways are under evaluation to improve clinical outcomes. HDTs restrict pathogen-induced subversion strategies to ameliorate host defenses against *M.tb*.

Futuristic therapeutics should aim to shorten the duration of conventional ATT by proficient elimination of persistent bacterial populations, which also result in drug-resistant strains. With the constant expansion of drug resistance, options for treatment are continuously depleting. Chiefly, in terms of drug-resistant TB, HDTs can be employed to augment antimicrobial host defences or to restrain detrimental inflammation instigated by infection. Some of the prospective HDTs against *M.tb* are listed in **Table 3** **, summarised in** **Figure 3** and further mechanism of protection is elaborated in different sections. Furthermore, HDTs that can limit the hepatotoxicity associated with conventional antibiotics are desired to subside unfavourable outcomes of extensive therapies. Theoretically HDTs surpass diverse issues associated with pathogen-directed therapeutics. HDTs augment host immune responses with sufficient proficiency to restrict the progression of the disease. Furthermore, targeting host components provide the advantage of reducing the generation of antibiotic resistance (129). Therapeutics targeting host components avoid the chances of drug resistance which is a global health concern. However, if not chosen wisely targeting host components can lead to off-target binding and might accelerate the chances of detrimental side effects. Thus, our knowledge regarding targeted mechanisms is vital to developing therapies to reprogram host defences for efficacious TB treatment.

**Table 3:** Potential host directed immunotherapeutic approaches against *M.tb* infection.

S. No.	Therapeutic candidates	Host protective immunological characteristics	References
**1.**	Ibuprofen	Inhibits neutrophil infiltration and detrimental inflammation at the site of infection	(108, 109)
**2.**	Acetylsalicylic acid (Aspirin)	Anti-inflammatory responses reduce detrimental pathology	(108)
**3.**	Monosodium Urate (MSU)	Activation of immune responses to augment antimycobacterial efficacy of BCG	(110)
**4.**	Calcimycin	Induction of autophagy by binding to P2X7 receptors	(111)
**5.**	Verapamil	Inhibits LTCC channels thereby induces autophagy by increasing Ca^2+^ levels.	(112)
**6.**	Clofazimine	Enrichment of stem cell memory T memory responses upon BCG revaccination	(113)
**7.**	Luteolin	Inhibition of Kv_1.3_ K^+^ channels, enhancement of antimycobacterial and T cell memory immune response	(114, 115)
**8.**	Rapamycin (Sirolimus)	Enhances antigen processing and presentation and directs T_h_1 immunity	(116)
**9.**	Tat-beclin-1 fusion peptide	Autophagy induction and reduction in progression of pathogens	(117)
**10.**	Gefitinib	Enhances lysosomal biogenesis, action and bacterial degradation	(118)
**11.**	2-deoxyglucose (2-DG)	Metabolic reprograming induced reduction in pathological damage	(119)
**12.**	Ritonavir (Norvir)	Glucose transporter agonist induces protection against HIV as well as *M.tb*	(120)
**13.**	FX11	Lactate dehydrogenase inhibitor reduces oxidative stress and downgrade iNOS	(121)
**14.**	TEPP46	Limits inflammation by reducing PKM2 activation	(122)
**15.**	Metformin	Induces AMPK mediated signaling, induction of ROS and intracellular bacterial killing	(123)
**16.**	AICAR	Stimulate anti-microbial immune responses by *via* (PPARGC1) linked pathways	(124)
**17.**	C75	Inhibits lipid derived droplets biogenesis, enhances ROS, NO production and polarizes macrophages from M1 to M2	(125)
**18.**	Cerulenin	Inhibition of fatty acid synthase, uncouples UCP2 and promotes NLRP3 activation	(126)
**19.**	GW9662	PPARγ antagonist can regulate inflammation and disease progression by altering metabolism in macrophages.	(127)
**20.**	AGK2	Inhibits host sirtuin2 (SIRT2) and enhances bacterial clearance, host protective immune responses	(128)

## Established Host-Directed Strategies to Potentiate BCG Vaccination

In an attempt to achieve the targets of End TB strategy, diverse host-directed therapeutics with the potential to reprogram host defences for better clinical outcomes are under consideration. Since it is known that in the majority of individuals, the immune system can self-reliantly eradicate the pathogen. Augmenting host defences so as to achieve complete sterility can offer benefits in reducing existing global TB burden. To achieve superior effectiveness against *M.tb* infections, researchers have evaluated the administration of immunomodulators along with antibiotics and vaccines. This was widely employed in cancer therapies wherein inhibition of anti-inflammatory cytokines, and inhibitory signaling receptors such as PD-1 and CTLA-4 were found effective in augmenting tumor recession (130). Likewise, therapeutics known for inhibition of detrimental immune responses were found effective in improving tumor vaccine efficacies. Constructive outcomes were detected against breast cancer and pancreatic adenocarcinoma by the utilization of COX-2 inhibitors (131). In HIV-infected individuals, therapy with COX-2 inhibitor augmented effector and memory responses induced by T cell targeting vaccine; tetanus toxoid (132). Selected clinical trials that have evaluated immunomodulatory strategies adjunct to BCG immunization for improved clinical results have been listed in **Table 4** (133). Furthermore, modulation of monocytic cell populations has been exploited as a prospective strategy for augmenting efficacy of BCG vaccination. Abundance of uric acid crystals namely monosodium urate (MSU) have been linked with bone inflammation and associated immune responses (110). Presence of MSU crystals was further linked with coexisting *M.tb* joint infection in patients suffering from gout (134). MSU treatment in the THP-1 cell line brings about a generation of ROS, stimulation of phagosome-lysosome fusion, and *via* NOD-like receptor signaling enhanced BCG clearance (135). MSU alone has no anti-bacterial activity inferring potential to promote bacterial clearance by immunomodulation. MSU therapy in adjunct to BCG vaccination *in vivo* led to a reduction in bacterial burden in draining lymph nodes. However, MSU treatment did not affect the viability of BCG. As compared to BCG alone, MSU therapy significantly reduced the bacterial burden in the lungs and spleens of *M.tb* infected mice (110). Based on affirmative evidences of Vitamin D supplementation in restraint of *M.tb* infections (136), several research groups have correlated protective efficacy of BCG immunization in infants with Vitamin D levels (137). One of the research group has observed increase in Vitamin D levels in infants vaccinated with BCG and have linked the upsurge with non-specific immune responses detected subsequent to vaccination (138). In another study, infants supplemented with Vitamin D were expected to elicit protective IFN-γ responses against *M.tb* infection (137). However, this clinical trial waned to provide evidences of Vitamin D induced protection in augmenting BCG efficacy. Further assessment is requisite to explore prospects of Vitamin D supplementation along with BCG immunization. Potential of established immunomodulators with BCG have been improvised for better clinical outcomes. One such study demonstrated induction of superior host protective T cell responses upon co-administering curcumin nanoparticles along with BCG immunization in murine model (139). Clofazimine (CLOF), an authorised therapeutic for leprosy treatment and second-line drug used in combinations against drug-resistant *M.tb* strains has also demonstrated affirmative outcomes in mice model. BCG revaccination along with CLOF administration significantly augmented T cell memory responses comprising enhancement of stem cell-like memory T cell responses (T_SCM_) along with successive effector and memory T cell populations (113). So as to further resolve the prospects of above-mentioned strategies in enhancement of BCG efficacy, further studies in higher animal models such as non-human primates (NHPs) and clinical trials is necessitated without delay.

**Table 4:** List of clinical trials evaluating BCG immunization along with diverse immunotherapeutic for efficient medical utility against diverse disease conditions.

BCG vaccine and immunotherapeutic regimen	Clinical trial	Trial identifier	Diseased condition	Ref.
Intravesical hyaluronic acid (HA) with BCG	Phase 2, randomized, pilot study to examine effect of HA in reducing BCG induced local cytotoxicity	NCT02207608	Bladder urothelial cell carcinoma	(12)
Tislelizumab in Combination with BCG	Phase 2, open-label, single-arm, single center trial to evaluate safety and effectiveness of Tislelizumab along with BCG (TACBIN-01)	NCT04922047	High risk urinary bladder cancers	(13)
Vitamin D supplementation in adjunct to BCG immunization in infants	Randomized, double masked, interventional study to evaluate impact of vitamin D supplement in infants prior to BCG vaccination	NCT01288950	Tuberculosis	(14)
Vitamin A with BCG Vaccine	Phase 4, randomized, double-masked intervention to evaluate the utility of high-dose vitamin A supplementation in infants along with BCG vaccine at birth	NCT00168597	Mortality and morbidity in infants	(15)
Monoclonal Antibody A1G4 and BCG	Phase 1 intervention to evaluate the efficacy of monoclonal antibody A1G4 along with BCG in cancer patients	NCT00003023	* Neuroblastoma, Sarcoma	(16)

## Modulation of Immune Responses to Improve Protective Efficacy

With the rise of genomics, researchers have utilized the genomic information of *M.tb* and *M. bovis* BCG vaccine strains for assessing variations that can be benefitted to develop better vaccination strategies (95). The revelation of the entire *M.tb* genome revolutionized TB research by expanding the knowledge of central immunomodulatory components (140). To achieve superior effectiveness against *M.tb* infections, researchers have evaluated the administration of immunomodulators along with vaccines. This was widely employed in cancer therapies wherein inhibition of anti-inflammatory cytokines, and inhibitory signaling receptors such as PD-1 and CTLA-4 were found effective in augmenting tumor recession (130). Likewise, therapeutics known for inhibition of detrimental immune responses were found effective in improving tumor vaccine efficacies. Constructive outcomes were detected against breast cancer and pancreatic adenocarcinoma by the utilization of COX-2 inhibitors (131). In HIV-infected individuals, therapy with COX-2 inhibitor augmented effector and memory responses induced by T cell targeting vaccine; tetanus toxoid (132). Inhibition of neutrophil infiltration at the site of *M.tb* infection by **Ibuprofen** (IBP) resulted in improved clinical outcomes and a reduction in bacterial burden in C3HeB/FeB mice (109). IBP is also known to possess specific antitubercular characteristics (141). Furthermore, IBP along with another drug; **acetylsalicylic acid** was evaluated to be repurposed as an adjunct therapy in TB patients (108). In murine model of TB, drugs were associated with enhancement of pyrazinamide (PYZ) antimycobacterial efficacy (142). This approach can be further exploited to amend BCG-induced responses. These studies indicate that treatment with immunomodulatory compounds or enriching host protective responses along with BCG might can effectively enhance protection induced by BCG vaccination.

## Targeting Host Ion Channels

Another strategy is to aim at host ion channels, that orchestrate physiological features of various cell populations by operating ions facilitated currents throughout cellular and subcellular membranes (143). Intracellular calcium levels are known to regulate key immune responses in the host, directly or by directional alteration of other vital ions such as potassium (K^+^), sodium (Na^+^), and chloride (Cl^-^) ions within immune cell populations (144). Obstruction of ion channels by employing diverse blockers has been assessed as a therapeutic target for diseases like hypertension (145). Research groups have also examined ion channel blockers for boosting anti-microbial immune responses. Intracellular calcium (Ca^2+^) levels play a vital role in the regulation of antimycobacterial mechanisms such as autophagy, maturation of phagosome, and induction of apoptosis (146). However, the impact of Ca^2+^ levels on processes like autophagy additionally depends on the involvement of diverse ion channels contributing to the maintenance of current (147). For instance, it has been observed that Ca^2+^ currents *via* Voltage-gated calcium channels (VGCCs) impede induction of autophagy (147) while Ca^2+^ currents through P2X purinoceptor 7 (P2X7) receptor heighten autophagy induction and intracellular extermination of *M. bovis* BCG in macrophages. **Calcimycin,** an ionophore binds to P2X7 receptor which leads to rise in intracellular Ca2^+^ levels and exerts antimycobacterial activity against *M. bovis* BCG (111) by stimulation of autophagy (148). Administering Ca2^+^ ion channel blockers was linked with a 32% reduction in risk of progression into a diseased state, in a clinical study in TB patients with heart and cerebrovascular diseases (149). Diverse Ca^2+^ ion channel blockers evaluated in the investigation exhibited variable consequences. L-type calcium channel (LTCC) blocker – **verapamil,** an FDA approved drug is utilized to treat abnormalities in heart rhythms, angina (150), and hypertension (151). In macrophages, LTCCs attenuate Ca^2+^ discharge from the endoplasmic reticulum (ER) leading to inhibition of macrophage activation. So as to bypass host immune responses *M.tb* upregulates the expression of VGCCs in APCs. Verapamil administration inhibits LTCC currents thereby escalating Ca^2+^ concentration in the cytosol which upregulates autophagy and bacterial clearance in *M.tb* (143). Additionally, LTCC ion channel blockers can alter iron-associated metabolic pathways thereby impeding iron accessibility which lowers intracellular bacterial survival (143). Furthermore, verapamil acts synergistically with first-line anti-TB drugs- INH (152), and RIF (153) in lowering the bacterial burden in cultures, macrophages, and murine models of TB. In another study, antimycobacterial activity of verapamil was confirmed with several TB drugs including bedaquiline (BDQ) and clofazimine (CFZ) and decreased bacterial load was linked with induction of membrane stress responses as a consequence of verapamil induced membrane function disruption (154). In the murine model, adjunctive verapamil administration augmented efficacy of recently approved MDR-TB drug; bedaquiline at lower doses and diminished the emergent resistant strains (155). Furthermore, progressive approaches such as assessment of inhalable verapamil-rifapentine particles has been assessed by researchers for utility as ATT (156). Even with significant pieces of evidence regarding anti-TB activity and negligible toxicity, verapamil has not transitioned into clinical setup. Further assessment of the anti-TB potential of verapamil along with BCG immunization is necessitated to evaluate the impact on modulating immunological responses. Similarly, Numerous K^+^ ion channel blockers have been assessed as prospective anti-TB therapeutics owing to their physiochemical ability to activate macrophages. **Clofazimine** (CFZ) is a conventional first-line drug used for leprosy treatment along with RIF and dapsone (157). It was initially developed against *M.tb* but was found not as efficacious as INH and RIF. However, with emergent drug-resistant strains, it has been recently employed as a second-line anti-TB drug (158). Numerous clinical trials (BEAT-TB (159), endTB-Q (160), and TB-PRACTECAL (161)) are investigating the efficaciousness of regimens comprising clofazimine. Furthermore, phase-2 clinical trial CLO-FAST is examining 3-month ATT comprising clofazimine and rifapentine against drug-sensitive *M.tb* (162). In addition to antimycobacterial activity, clofazimine is a potent immunomodulator. It inhibits Kv_1.3_ K^+^ channels which are expressed in various immune cells (163). Clofazimine-induced inhibition of Kv_1.3_ K^+^ channels abundantly present on T effector memory cells (T_EM_) enhances the efficacy of BCG vaccination in a murine model of TB by specifically promoting the expansion of the T central memory cell population (T_CM_). Furthermore, CFZ enriches stem cell memory T cell responses upon BCG revaccination (113). In a similar manner, we have demonstrated the efficacy of less toxic, phytochemical namely Luteolin an established Kv_1.3_ K^+^ channel blocker in augmenting BCG-induced immune responses by enriching T_CM_ memory responses, improving T_CM_ : T_EM_ ratio and enhances host protective T_h_1 and T_h_17 immune responses against *M.tb* infection in the murine model of TB (115). Furthermore, immune-protective properties of luteolin condensed the time period of bacterial clearance with INH owing to augmented T_h_1 and T_h_17 immune responses and eased pathological damage and TB associated hepatotoxity *in vivo* (114).

## Improving Anti-Microbial Immune Responses

Therapeutic modulation of host immune responses to achieve complete sterility is another approach that has been pursued by several research groups (129). *M.tb* utilizes complex artillery to evade immune cell populations and associated defense mechanisms. *M.tb* owing to mycobacterial virulence factors such as cell wall component- mannose-capped lipoarabinomannan is known to inhibit phagolysosome fusion in macrophages. It is a vital phenomenon critical for curbing infection at an early stage (26). However, this can be enforced by autophagy induction, which is another cellular mechanism by which detrimental cytosolic molecules and organelles are targeted to lysosomes for degradation. Early secreted antigen 6 secretion system-1 (ESX-1) of *M.tb* is known to permeabilize the phagosome to escape degradation (164). However, this facilitates processing by components of ubiquitin-mediated autophagy mechanism and results in a reduction in *M.tb* persistence (165). Furthermore, stimulation of autophagy sequesters and degrades bacterial components and can also contribute to fostering antigen presentation and moderating pathology (166). The most widely studied autophagy inducer – **rapamycin (Sirolimus)** is known to inhibit the mammalian target of rapamycin (mTOR) which negatively regulates autophagy. It is majorly employed in organ transplantation owing to the immunosuppressive nature of the drug (167). Researchers have attempted re-purposing of Rapamycin, an autophagy inducer to heighten antigen processing and presentation in murine antigen-presenting cells (APCs) (168). It is well established that confiscation of BCG within phagosome and inability to fuse with lysosome reduces the efficacy of antigenic peptide presentation on DCs. Dendritic cells (DCs) treated with autophagy inducer, rapamycin enhanced T_h_1 responses against *M.tb* (116). Improvement in DC activation and T_h_1 responses against *M.* with concurrent rapamycin and BCG administration offers prospective approach to autophagy mediated enhancement of bacterial clearance (116). Similarly, the efficacy of BCG can be augmented by simultaneous treatment to direct host responses toward bacterial extermination to achieve complete sterility (116). Few piecemeal studies have questioned the immunotherapeutic strategies to enhance vaccine efficiency against TB, however a great deal is yet to be explored (169). However, side effects associated with rapamycin administration such as interstitial pneumonitis can be alarming in TB patients with substantial pathology (170). Furthermore, metabolization of rapamycin by hepatic enzyme CYP3A4 limits its utility in TB patients since CYP3A4 is intensely stimulated by standard ATT antibiotic – INH (171). Due to the mentioned limitations rapamycin has not been further evaluated as HDT against *M.tb*. **Vadimezan (also known as DMXAA)** is another prospective autophagy inducer. It is an established antitumor agent as well as in mice it triggers, a stimulator of IFN genes (STING) dependent autophagy mechanism (172). However, it was found inefficacious in humans (173). Research groups have further examined the utility of fusion peptides for the induction of autophagy. **Tat-beclin-1 fusion peptide** an autophagy inducer (174) was found to restrict the proliferation of diverse pathogenic strains and heightened survival rates in infected mice (117). Though, restrictions like regular administration by injection constrain the clinical utility of HDT. Alternatively, an inhibitor of epidermal growth factor receptor (EGFR) was found to limit *M.tb* proliferation in macrophages and reduces bacterial burden in the lungs of infected mice *via* autophagy induction (175). In *M.tb* infected macrophages treated with **Gefitinib**; tyrosine kinase inhibitor, lysosomal biogenesis, function and targeting of bacteria to lysosome for degradation is increased thereby decreasing bacterial burden *via* EGFR signaling in macrophages (118). However, there is a need to further analyse pieces of evidence and peripheral markers of autophagy for certainty. With the advancement in technologies, sophisticated approaches to evaluate the induction of autophagy can evolve the quest for superior autophagy inducers that can be employed to enhance bacterial killing by limiting dissemination.

## Targeting Host Metabolism

Research focus has shifted radically in the past decade towards metabolic shifts in response to infections. Immunometabolism is an emerging field that focuses on the impact of the metabolic state of immune cell populations to provide better insight into disease progression and pathogenesis (176). In the initial course of *M.tb* infection, metabolic shift is observed to defend the host. Immune protective responses such as stimulation of pro-inflammatory cytokines, and nitric oxide (NO) release is directed *via* HIF-1-dependent glycolytic pathways (177). However, *M.tb* is known to stimulate the Warburg effect so as to inhibit anti-microbial host immune responses (178). Host metabolism is utilized by *M.tb* to survive and proliferate by escaping host protective immune mechanisms (179). This infers that metabolic reprogramming is vital for defense against *M.tb* so as to augment efficacious sterilization mechanisms. **2-deoxyglucose (2-DG)** an inhibitor of hexokinase enzyme, can limit the IL1-β generation in LPS-activated macrophages and result in succinate accumulation (180). 2-DG stimulated glycolysis inhibition can additionally result in a reduction in lung damage induced by LPS (181) *via* moderating nuclear PKM2-STAT3 signaling. Further, prospects of 2-DG to restrict pathological damage in TB cases can be assessed for augmented clinical outcomes. Similarly, **ritonavir** (Norvir), a protease inhibitor widely used as antiretroviral medication to treat HIV infections (120), is additionally known for capability to act as an glucose transporter agonist (182). Researchers have evaluated combinations of HIV drugs including ritonavir along with ATT so as to effectively counter HIV-TB coinfections (183). Strategic arrangement by utilizing characteristics of ritonavir to inhibit host glucose transporters can be assessed further in case of HIV-TB patients to better understand mechanism of protection. Inhibitor of pyruvate dehydrogenase kinase- **dichloroacetate**, is a small molecule that increases pyruvate flux into mitochondria and skews metabolism toward glucose oxidation rather than glycolysis (184). Inhibition of pyruvate dehydrogenase kinase was established as a host target to counter infection of *Salmonella enterica serovar typhimurium via* metabolic reprogramming of M1 macrophages. However, intracellular burden for *M.tb* was not reduced upon dichloroacetate treatment, alternate inhibitors can be explored with similar objective (185). Another small molecule and lactate dehydrogenase inhibitor – **FX11 (**3-dihydroxy-6-methyl-7-(phenylmethyl)-4-propylnaphthalene-1-carboxylic acid]) is known for induction of oxidative stress and reduction in tumour advancement (121). Downregulation of iNOS and cytokine generation was achieved in LPS-activated RAW 264.7 macrophages upon FX11 induced lactate dehydrogenase inhibition (186). Similarly, the inhibitor of pyruvate kinase– **TEPP46** significantly reduced PKM2 activation in LPS-induced macrophages which led to a lowering of IL-1β generation (122). Hence, small molecule inhibitors can be employed to direct metabolic flux for desired clinical outcomes to resolve immunopathology of TB by regulating host metabolism.

*M.tb* manipulates lipid and fatty acid metabolic pathways of the host for its persistence and proliferation (187). Foamy macrophages recruited around *M.tb* infected phagocytes, supply nutrition and support in the course of infection. *M.tb* manipulates host cells to synthesize lipids and fatty acids. Hence, components of lipid synthesis pathways manipulated by *M.tb* for survival can be targeted as HDT (188). Metabolic energy sensors such as AMP-activated protein kinase (AMPK) play a vital role in the regulation of key host protective mechanisms against infections (189). An approved type 2 diabetes drug, **Metformin** which activates the AMPK-mediated signaling mechanism has been evaluated for TB (190). Metformin induces the generation of mitochondrial reactive oxygen species (ROS) resulting in restriction in intracellular growth of *M.tb* and limiting the activation of the inflammatory gene (191). In *M.tb* infected guinea pigs, metformin acts synergistically with conventional ATT drugs – INH and ETH (192). Metformin administration causes a significant reduction in latent TB incidences in prone diabetic individuals (193). This HDT can be evaluated proficiently at advanced clinical stages. Another AMPK activator, 5-aminoimidazole-4-carboxamide-1-1-β-D-ribofuranoside **(AICAR)** stimulates anti-microbial responses by activating autophagic pathways in macrophages. AMPK activation by AICAR further controls the biogenesis of mitochondria and metabolic state in macrophages by inducing peroxisome proliferator-activated receptor gamma coactivator-1 (PPARGC1) associated pathways (124). Components of host machinery that curb the metabolism of lipids can reduce detrimental inflammation thereby establishing a balanced immune state. Fatty acid synthase inhibitors such as **C75** and **cerulenin** are prospective targets for the augmentation of efficacious immune responses. Inhibition of lipid-derived droplets by C75 can lead to polarization of macrophages from the M1 to M2 subset causing enhancement of ROS and NO production (125). C75 and cerulenin-mediated inhibition of fatty acid synthase lead to uncoupling protein-2 (UCP2) mediated NLRP3 inflammasome activation (126). PPARγ antagonist, **GW9662** is known to regulate vital processes such as metabolism, inflammation, and disease progression (127) in macrophages infected with *M. bovis* BCG (76). Link between inflammation and lipid metabolism associated PPARγ signaling can be exploited to potentiate BCG-induced protection (76). This infers that reprogramming key components of lipid metabolism can be a prospective target for progressive TB therapeutics. Sirtuins (SIRTs) are another prospective target with the potential to be targeted for the augmentation of host defences (77). SIRTs are deacetylases that regulate cellular mechanisms like inflammatory responses, regulation of lipid metabolism by modulating components of NF-κB immune signaling, and anti-inflammatory responses by regulation of Peroxisome proliferator-activated receptor gamma coactivator 1-alpha (PGC-1α) (78). It has been observed that SIRT-1 expression is diminished drastically in *M.tb* infected THP-1 cells. SIRT-1 downregulates RelA/p65 unit of NF-κB so as to modulate inflammation (79). SIRT-6 is also known to diminish pro-inflammatory and anti-microbial responses in the early course of *M.tb* infection (80). Furthermore, SIRT2 has been established as an immunotherapeutic target against *M.tb* infection in mice model of TB (128). It was observed that subsequent to *M.tb* infection, SIRT2 expression increases along with translocation to nucleus to induce immune dampening epigenetic modifications. However, chemical inhibition of SIRT2 using established inhibitor, **AGK2** dramatically augmented bacterial clearance and host protective immune responses. Since, existing literature is available regarding SIRT2 induced metabolic programming (81) and signal transduction (82). Further progressive approaches can be shaped by targeting key physiological factors of host.

## Targeting microRNAs

miRNAs are non-coding RNAs that are involved at post-transcriptional levels to regulate array of genes decisive of immune responses (83). Over 2000 functional miRNAs are encoded by human genome (84) which regulate diverse protein-coding transcripts (194). It is now well-established that miRNAs distinctly regulate host immune responses against *M.tb* infection (195). Differential expression of miRNAs can signify the advancement of disease from latent to an active infection (196). Furthermore, miRNAs play a significant role in the moderation of apoptotic and autophagic responses during *M.tb* infection (196). Owing to advancements in technology, miRNA delivery is being employed to treat diverse diseases. This further pave way for the application of miRNAs as HDT against TB (197). Several studies have assessed mechanisms by which *M.tb* temper host immune responses for survival in an antimicrobial milieu. Diverse immune mechanisms such as phagolysosome maturation in APCs, cytokine stimulation by immune cell populations, and antigen processing and presentation are dynamically manipulated by *M.tb*. These cellular processes are strictly regulated by an assortment of miRNAs in the host (198). *M.tb* is additionally known to alter miRNA expression associated with key biological responses to escape host immune responses (199). Additionally, expectations of combined regulation of transcriptional network by miRNAs and transcription factors represent miRNAs linked with diseases as a novel category of therapeutics.

miRNAs involved in immune pathways are extensively studied for mycobacterial infections (200). It is well-known that during *M.tb* infection miR-125b inhibits TNF biosynthesis in human alveolar macrophages (201). Several research groups have observed that *M.tb* infection results in differential expression of miRNAs which determines the fate of immune responses (202). However, comprehensive knowledge is requisite for progressive considerations. Participation of miRNAs in *M.tb* induced autophagy (203) and apoptosis has provoked further interest to maneuver miRNAs for HDTs. Upon *M.tb* infection, diverse cell populations respond varyingly in conjunction with variations in miRNA expression. Upregulation of miR-155 has been observed in bone marrow-derived macrophages of mice infected with *M.tb* (204). In contrast, downregulation of miR-155 has been observed in peripheral blood mononuclear cells (PBMCs) derived macrophages upon *M.tb* infection (201). Mycobacterial infection in human monocyte-derived macrophages results in overexpression of miR-29a, miR-886-5p, and let-7e, which further target caspase 3 and caspase 7 as predicted by the integrated analysis. Differential expression of miR155, miR-146a, miR145, miR222, miR-27a, and miR-27b was observed in human macrophages infected with virulent *M.tb* H37Rv and avirulent strain *M. bovis* BCG. Downregulation of miRNA involved in the regulation of inflammation and lipid metabolism was observed. Furthermore, miR-145 known for induction of apoptosis has been reported to be downregulated upon infection with a virulent *M.tb* strain, which results in overexpression of targets which inhibit apoptosis (205). Based on microarray analysis, global changes in miRNA expression screened nine miRNA genes which were differentially expressed in *M.tb* H37Rv and *M.tb* H37Ra infected THP-1 cells. These differentially expressed miRNAs such as miR-30a, miR-30e, miR-155, miR-1275, miR-3665, miR3178, miR-4484, miR-4668-5p, and miR-4497 contribute in diverse physiological aspects. miR-155 interacts with negative regulators involved in TNF- α generation (201). Evaluation of PBMCs and pleural fluid mononuclear cells (PFMCs) linked miRNA expression with levels of IL-6 cytokine. Additionally, it has been observed that the highly-virulent Beijing/W TB strain represses plenty of miRNA in human macrophages as compared to non-Beijing/W TB strains. Alterations in miRNAs have been observed in patients with active TB. The functional assessment demonstrated that miR-144 restrains T-cell expansion and generation of key cytokines, INF-γ and TNF-α. In RAW264.7 cells, upon BCG inoculation, miRNA-144-3p overexpression is linked with inhibition of autophagy and antimycobacterial activity (206). Elevation in miR-424 and miR-365 levels has been detected in active TB patients. Diverse miRNA contributes to determining the fate of infection. Regulation of immune cell activation by miR-155, miR-146a, miR-21, and miR-9 (207), TLR signaling is positively regulated by miR155 (208). Subsequent to *M. bovis* BCG infection significant increase in miR-155 expression is observed in macrophages, which modulates diverse innate immune responses including ROS generation (209). It additionally plays role in apoptosis induction in macrophages upon *M. bovis* BCG inoculation which modulates cellular physiology and immune responses (210). In *M.tb* infected human alveolar macrophages, miR-125b inhibits TNF generation (201). miR-29 targets IFN-γ and regulate immune responses linked to *M.tb* infection (211). miR-223 targets several chemo-attractants such as CXCL2, CCL3 and contributes to directing immune response (212). It has been evaluated that endogenous block of miR-29 in transgenic mice, augmented resistance to *M.tb* infection (211). miR-27a targets IRAK4 and restrict immune response in TB (213). Another study demonstrated that BCG infection in RAW264.7 cells upregulates miR-17-5p which was linked with augmented BCG dissemination and enhanced autophagosome related protein expression (214). As revealed diverse miRNAs are under evaluation owing to gene modulatory potentials. However, our knowledge regarding role of specific miRNA overexpression upon BCG inoculation is still fragmentary. Utilization of miRNAs to augment host immune responses, paves way for advancement in therapeutics for various diseased conditions. Differential expression of miRNAs can be moulded in such a way to achieve better clinical outcomes in TB patients. Progressive therapeutics are employing miRNA-mimics (215), antisense oligonucleotides (216) to manipulate immune responses. Although several research groups have evaluated the utility of miRNA manipulation as HDT against *M.tb*, further assessment is necessitated to establish the prominence of miRNA as therapeutic. We required more studies to comprehend the contribution of miRNA in host-pathogen interactions and a progressive strategy for augmenting conventional therapies.

## Conclusions and Future Perspective

Despite incessant debates on the variable protective efficiency of BCG, it prevails as the only vaccine for TB prevention. Owing to considerable protection in children against disseminated forms of TB, it remains a key component of TB control programs in various countries (90). Throughout our review, we have mentioned several shortcomings and lacunae linked with the failure of the TB vaccination strategy. One of which is the complexity of the disease itself, as we are envisaging resolutions that can impart complete sterility, which is seldomly accomplished in the natural course of infection (26). In this aspect, TB diverges from the diseases that are preventable *via* vaccination. Hence, progressive approaches were pursued with the utilization of known immune correlates of protection. To surpass the previously failed attempts, it is vital to redirect focus on immunological pathways that can be augmented for better clinical implications (217). Since adult pulmonary TB mainly accounts for *M.tb* transmission, advances to tackle the inadequacies of BCG to impart long-lasting immunological memory should be highlighted as a better immunization stratagem. With extraordinary scientific efforts to counteract the most fatal pathogens known to humankind, we have progressed to a situation wherein we can harness establish knowledge and immunological concepts to deal with the shortcomings of existing approaches and improvise for robust clinical trajectories. Since the most of individuals worldwide are already vaccinated with BCG, it is judicious to keep BCG in reflection while developing new vaccination strategies. Scientific communities are attempting stratagems to prevent millions of deaths from escalating infectious diseases by investing on immunotherapeutic approaches to augment immunological responses.

## Author Contributions

KN, VD wrote the manuscript. AB edited the manuscript. VD conceived the hypothesis. All authors contributed to the article and approved the submitted version.

## Conflict of Interest

The authors declare that the research was conducted in the absence of any commercial or financial relationships that could be construed as a potential conflict of interest.

## Publisher’s Note

All claims expressed in this article are solely those of the authors and do not necessarily represent those of their affiliated organizations, or those of the publisher, the editors and the reviewers. Any product that may be evaluated in this article, or claim that may be made by its manufacturer, is not guaranteed or endorsed by the publisher.
